# Less invasive management of deep neck infection and descending necrotizing mediastinitis: A single-center retrospective study

**DOI:** 10.1097/MD.0000000000006590

**Published:** 2017-04-14

**Authors:** Dong Wei, Ling Bi, Huiyong Zhu, Jianfeng He, Huiming Wang

**Affiliations:** Department of Oral and Maxillofacial Surgery, The First Affiliated Hospital, School of Medicine, Zhejiang University, Hangzhou, China.

**Keywords:** deep neck infection, descending necrotizing mediastinitis, emergency surgery, less invasive surgery

## Abstract

By a 7-year retrospective review, we reported our experience in management of descending necrotizing mediastinitis (DNM) and deep neck infection (DNI). A retrospective design was used to reveal the clinical characteristics of DNI and DNM. The clinical outcome was analyzed to validate less invasive management. We reviewed 82 patients between 2009 and 2016, 12 of which were diagnosed as DNM by clinical and computed tomography findings. A total of 35 patients had relevant systemic conditions, mainly diabetes mellitus (19 patients). Most cases were secondary to oropharyngeal or dental infections. All patients underwent transcervical drainage, and 10 DNM patients were treated with additional closed thoracic drainage simultaneously. Twenty patients accepted more than 1 operation. Seven patients died as a result of sepsis and/or multiple organ failure. The mortality rate in our study was similar to that in other studies. In our opinion, less invasive therapies are useful to most patients. Transcervical drainage alone is optimal management for all DNI cases and some DNM cases. Additional closed thoracic drainage is enough for type I and IIA DNM with pleural effusion or empyema.

## 1 Introduction

Despite the wide use of antibiotics, great progress in critical care treatment, and surgical drainage, the mortality from deep neck infections (DNIs) of oropharyngeal and dental origin are still high. Delay in the diagnosis and treatment of DNI may result in grave consequences. Once patients with DNI complicated by descending necrotizing mediastinitis (DNM), one of the most disastrous complications, the mortality rate may be as high as 60%. Most surgeons believe that early diagnosis and invasive treatment are mandatory to effectively control the disease.

Surgeons have not reached a consensus on the standard treatment protocol for such patients due to diversity in the causes and locations of infection. Some DNI cases need surgical intervention, usually cervical drainage. Almost all cases developing DNM require some kind of drainage or debridement. But the optimal technique of drainage and the most suitable surgical approach remain debatable. We report the clinical results of DNI group compared with DNM group. Special focus was put on the clinical features and risk factors of DNI progressing to DNM. Our study will be helpful to a deeper understanding of this serious disease.

## 2 Methods

The study was performed in accordance with the guidelines of the Helsinki Declaration of 1975, as revised in 1983. It was approved by the ethics committee of the First Affiliated Hospital, School of Medicine, Zhejiang University. For this type of study formal consent is not required.

We retrospectively reviewed all cases of DNI including DNM that were treated at the Department of Oral and Maxillofacial Surgery, the First Affiliated Hospital, School of Medicine, Zhejiang University (between 2009 and 2016). Clinical charts, imaging, etiologies associated with systemic diseases, bacteriology, infectious origin, and duration of hospitalization as well as clinical outcomes were reviewed.

In the present study, 82 patients were suffering from DNI, 12 of which were diagnosed as DNM by clinical and computed tomography (CT) findings according to the criteria defined by Estrera et al.^[1]^ The relationship between oropharyngeal or cervical infection and DNM was clearly established. The 82 patients with DNI were divided into group I (without DNM) or group II (with DNM). According to classification of DNM defined by Endo et al,^[2]^ 10 cases in DNM group were type I, another 2 were type IIA.

Continuous variables (age and length of inpatient treatment) in this study were reported as mean ± SD. Categorical variables were presented as proportions. Different variables were compared between group I and group II. Unpaired Student *t* tests were used for measurement data. χ^2^ test was used for enumeration data. The level for statistical significance was defined as *P* < .05. All analysis was done using IBM SPSS Statistics, version 19.

## 3 Results

**Figure 1:**
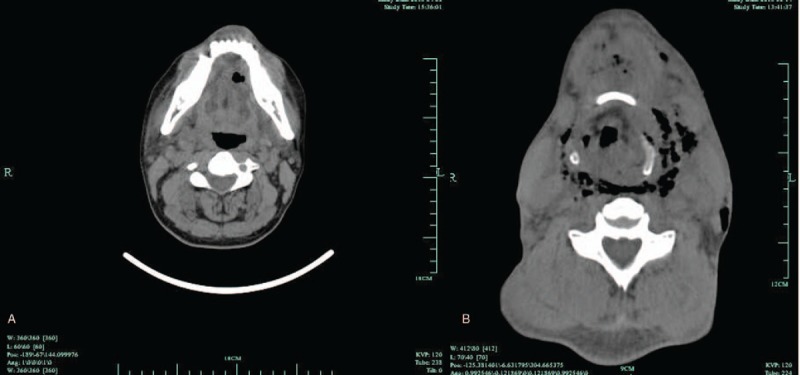
(A) Computed tomography (CT) scanning showed the formation of a left sublingual abscess following first molar periodontitis. (B) CT scanning showed abscess formation and gas production in parapharyngeal and submental spaces.

During the 7-year period in which the clinical data were collected, our department totally admitted 82 patients for DNI. Of them, 56 patients (68.3%) were male and 26 (31.7%) were female. The patients’ age ranged from 22 to 75 years (mean: 50.3 ± 12.8 years). The clinical data were recorded (Table 1). We found most patients had been given intravenous antibiotics before admission. Some patients had relevant systemic conditions including kidney failure, neutropenia, and diabetes mellitus (DM). On admission routine blood tests revealed an increase of white blood cells (16.69 ± 3.22  × 10^9^/L) in most patients (n = 76). The elevated values of C-reactive protein (31.25 ± 14.28 mg/L) were also found (n = 79). CT imaging showed signs of DNI in all patients. Oropharyngeal and dental infections were the 2 most frequent causes (42.7% and 37.8%) (Fig. 1). CT imaging revealed the extension of infection with pus descending into the mediastinum in 12 patients. The diagnosis of DNM was confirmed in subsequent surgical procedure. Typical signs of DNM were recorded in these 12 patients and included chest pain, jugular distension, high fever, and crackling on palpation. They also had symptoms of DNI, such as cervical pain, skin inflammation, upper airway obstruction, subcutaneous emphysema, and so on. The age, gender, symptoms, past history, infection dissemination, and course were shown in Table 2.

**Table 1:**
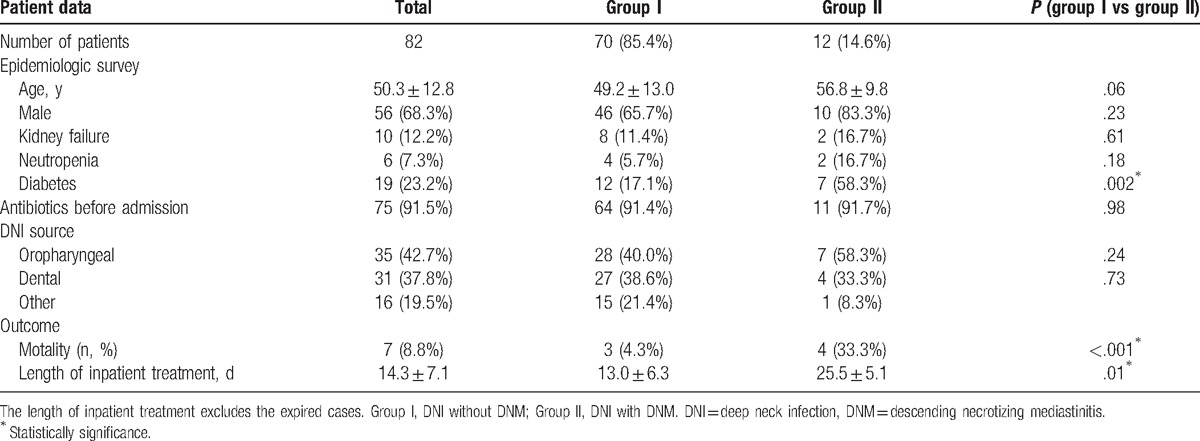
Patient history and clinical data.

**Table 2:**
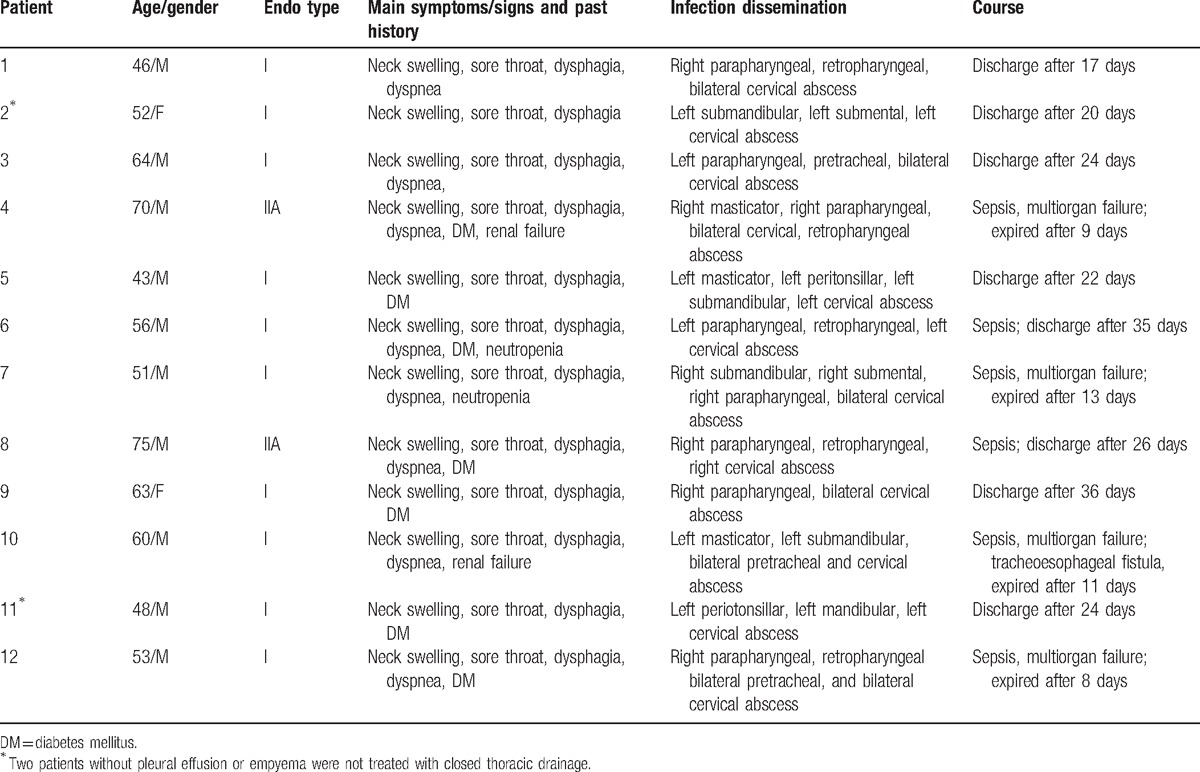
Summary for 12 patients with descending necrotizing mediastinitis.

Results from microbiological tests were obtained from 78 patients (95.1%). Examinations usually performed from blood samples and material obtained intraoperatively. Many patients showed a polymicrobial infection with mixed aerobic and anaerobic organisms (Table 3). *Streptococcus* spp were the most common aerobic and *Bacteroides* spp the most common anaerobic bacteria in our study.

**Table 3:**
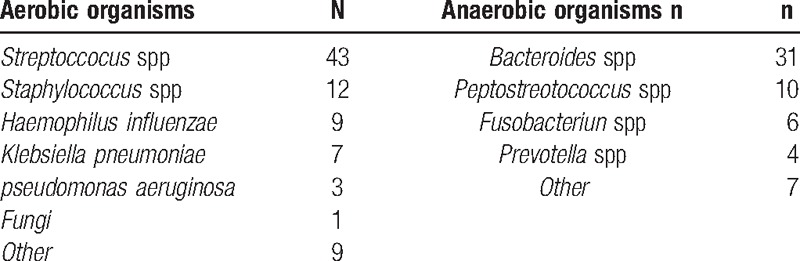
Results of microbial investigations (n = 82).

Antibiotic therapy was initiated on admission once the diagnosis was established. Empiric therapy usually began with 2nd-generation cephalosporins combined with metronidazole. Antibiotics were later altered based on the microbiological examinations and antibiograms.

**Figure 2:**
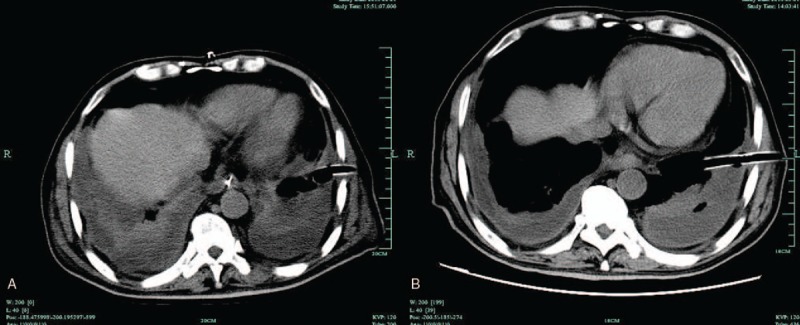
(A) Thoracic computed tomography (CT)-scan: multiperforated pleural drainage tube was positioned for patients with pleural effusion or empyema. (B) Thoracic CT-scan: effusion or empyema reduced observably after closed thoracic drainage.

Surgical treatment included elimination of the oropharyngeal or dental infection focus, and sufficient drainage applied to the neck and the mediastinum (Table 4). In this study, all DNI cases accepted transcervical drainage. Cervical abscesses were drained with a horizontal incision parallel to the superior border of clavicle. The submandibular abscesses were drained separately. We routinely positioned multiple Penrose drains in the submandibular and cervical region or just left the incisions open, irrigating the neck space with dilute povidone iodine solution twice a day. Undoubtedly, transcervical drainage was also the mandatory therapy for all DNM patients. Except transcervical mediastinal drainage, they were treated with additional closed thoracic drainage simultaneously if there was associated pleural effusion or empyema (n = 10). For closed thoracic drainage, we used multiperforated pleural drainage tubes, irrigating within the mediastinum or pleural cavity with saline (Fig. 2).

**Table 4:**
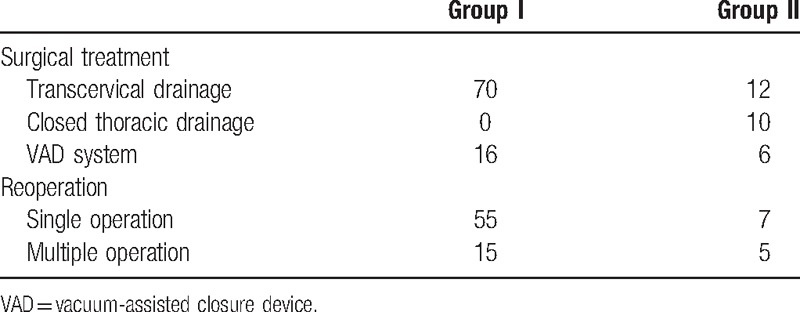
Summary of treatment.

All surgical procedures were performed under general anesthesia. The interval between hospital admission and the 1st operation ranged from 0 to 4 days. Repeated CT scanning in a 48 hours pattern was performed as a useful tool to assess the progression of abscesses and the need for further surgical management. A total of 35 patients (42.7%) underwent tracheotomy for deteriorated neck swelling or laryngeal edema. They were transferred to the intensive care unit immediately after the surgery.

A vacuum-assisted closure device was used in 22 cases as an auxiliary equipment which was helpful to tissue approximation and drainage of abscess. The vacuum-assisted closure device system was wrapped into the debrided cervical region and sealed the wounds with better air tightness. It was exchanged every 2 to 3 days. If necessary, redrainage and redebridement were performed during exchange procedure.

Seven patients died as a result of sepsis and/or multiple organs failure. One patient died of unexpected airway obstruction. The other 74 patients survived finally. Their length of inpatient treatment at our department ranged from 4 to 41 days. A total of 20 (24.4%) patients underwent more than 1 surgical procedure for repeated drainage and debridement. Complications were recorded in 34 patients (41.5%), including sepsis (n = 22), pleural effusion or empyema (n = 10), renal failure (n = 10), multiple organ failure (n = 8), and acute respiratory distress syndrome (n = 3).

## 4 Discussion

DNI remains a serious challenge for surgeons, especially when it develops DNM. DNM is a life-threatening infection and destruction of the mediastinal structures. Many research conclusions attribute its high mortality rate to difficulty in the diagnosis and treatment.^[3,4]^ Advent of antibiotics and modified surgical management only slightly decrease the mortality.^[5,6]^

The most common sources of DNI are pharyngeal and odontogenic infections. DNM secondary to DNI also derives from these origins frequently. There are more opportunities for DNI patients with a compromised immune system to suffer DNM. DM, kidney failure, neutropenia, and alcoholism are believed to be common predisposing factors for immune system deficiency.^[7,8]^ In our study, patients in group II had significantly high diabetic morbidity compared with patients in group I. We considered DM as a risk factor for DNM. In addition, reduced tissue oxygenation also plays an important role in the development of DNM. Consistent with many reports, we noticed that most microbiological tests showed a polymicrobial infection. This symbiotic relationship of aerobic and anaerobic organisms causes impaired tissue oxygenation synergistically, aggravating associated pathologies such as diabetes and respiratory insufficiency.^[8,9]^

Cervical and thoracic anatomy is closely related to the arising of DNM from DNI. There is an extensive fascial communication between the pharyngeal, gingiva, neck, and mediastinum.^[6,10]^ The retropharyngeal, vascular, and pretracheal spaces are quite vulnerable to the extension of infection. Downward spread of DNI is always accelerated by gravity, respiration, the negative intrathoracic pressure in the mediastinum, and pleural cavities.^[5,11]^

In this study, all patients suffered cervical pain. Some of them presented with lower cervical and upper thoracic soft tissue swelling and inflammation. In some cases, sepsis developed soon for releasing of bacteria and toxins into the circulatory system. For DNM patients, hyperpyrexia, progressive chest pain, jugular vein distension, and shortness of breath were typical manifestation. But DNM was hard to be differentiated from DNI by clinical manifestation only, which should be responsible for its delayed diagnosis and treatment.

The criteria for DNM diagnosis were defined in 1983, which emphasized demonstration of characteristic image features. For decades, a lot of authors have approved the significance of CT as the mainstay for early diagnosing of DNM.^[11–14]^ CT scanning is also an accurate and specific tool to direct surgical drainage and monitor postoperative progression. We considered contrast-enhanced CT as the “gold standard” in the diagnosis and evaluation of DNI and DNM. Many studies revealed that the typical signs of mediastinal infection on CT were unenveloped fluid collections, abscess, and soft-tissue gas infiltration. But widening of the mediastinal structures was the most common change in most cases.

For infections like DNI and DNM, antibiotics treatment is mandatory. But it is widely admitted that antibiotics alone cannot resolve DNI or DNM. Accordingly, a multidisciplinary approach including intensive care, aggressive surgical intervention, and simultaneous management of the comorbidities is recommended. It is no doubt that cervical drainage is indispensable for all DNI and DNM cases, but the standard treatment protocol has not been established yet.

Focus of the current controversy is on the surgical management for DNM. Many surgeons emphasize that the mediastinum cannot be drained adequately by a transcervical approach. They call for compulsory mediastinal exploration and debridement despite the involved level.^[11,15]^ But an increasing number of doctors will chose a more rational and individualized strategy according to clinical types.^[3,9,16]^ We were opposed to overtreatment, such as mediastinal exploration and thoracotomy. Excessive surgery may be harmful to DNM patients, especially for critically ill patients. Based on our own experience, we established a less invasive principle as below: transcervical drainage for all DNM; additional closed thoracic drainage for DNM with pleural effusion or empyema. We followed this protocol, achieving satisfying clinical outcomes.

The mortality rate of DNM in our study was 33.3%. It is important to note that the majority of DNM deaths were in the presence of severe illness, such as kidney failure, neutropenia, and DM. The mortality was similar to those in studies using more aggressive surgery. In other related studies, the reported mortality rate was still 20% to 50% despite of new advances in pharmacotherapy and surgical treatment.^[6,8,11,17,18]^ The rate could even increase to 67% in patients with severe systemic disease.^[4]^ Our protocol appeared to be workable. But we could not draw a conclusion about the clinical efficacy of our protocol for type IIB DNM because there was no type IIB DNM in our study.

In addition, the role of some other treatments remains debatable. Some doctors recommend tracheotomy and intubation after hospitalization for all patients because of possible respiratory insufficiency.^[3,6,12]^ But still some authors insist that tracheostomy be evaluated on an individual basis.^[7,15]^ They worry about tracheostomy sites to be a source of infection spread and subsequent mediastinitis. In some researches, hyperbaric oxygen therapy is believed to be useful to reduce mortality. But this treatment itself will disturb other procedures in the acute stage.^[9,15]^ The advantages of video-assisted thoracic surgery (VATS) are impressive, including reduced pain, less invasiveness, and faster recovery. Unquestionably, it provides a clear visual window to manage DNM. But it is still in dispute about the efficacy of VATS to achieve ideal drainage and irrigation for severe cases.^[18,19]^ Furthermore, there is a negative opinion that transthoracic approach for VATS should be avoided for DNM localized to the upper or anterior mediastinum. In such conditions, mediastinal abscess can be effectively drained by the transcervical approach.^[20,21]^ The role of steroids in the treatment of DNM is embarrassed for its antiinflammatory and immunosuppressive function. Some authors remind us that the use of steroids can be a risk factor for DNM onset and deterioration.^[22–24]^

## 5 Conclusion

The high mortality rate of DNI/DNM is still an embarrassment for many doctors. Excessively aggressive treatment is not necessarily favorable to the patients. Some less invasive therapies may also prove useful. According to our experience, transcervical drainage alone is optimal management for all DNI cases and some DNM cases. Additional closed thoracic drainage is enough for type I and IIA DNM with pleural effusion or empyema. We believe our study will be helpful to choose optimal treatment for DNI and DNM.
